# Non-sustained Ventricular Tachycardia in Children and Adult Congenital Heart Disease: Optimal Duration and Clinical Significance

**DOI:** 10.1007/s00246-025-04086-y

**Published:** 2025-11-13

**Authors:** Iqbal El Assaad, Lama Dakik, Abdel Rahman E’mar, Alison Heilbronner, Peter Aziz, Akash Patel

**Affiliations:** 1 Children’s Institute Department of Heart, Vascular & Thoracic, Division of Cardiology & Cardiovascular Medicine, Cleveland Clinic Children’s, Cleveland Clinic Lerner College of Medicine, 9500 Euclid Avenue, Cleveland, OH USA; 2 Cleveland Clinic Lerner College of Medicine, Cleveland, OH USA

**Keywords:** Non-sustained ventricular tachycardia, Congenital heart disease, Sudden cardiac death, Ambulatory monitoring, Children

## Abstract

The frequency and clinical significance of non-sustained ventricular tachycardia (NSVT) detected on ambulatory monitors in pediatric and congenital heart disease (CHD) patients (pts) has not been thoroughly investigated. This study aims to examine the prevalence of NSVT, analyze the presence of associated cardiovascular disease, and describe changes in management. We performed a retrospective cohort study of all pts evaluated in cardiology clinic at Cleveland Clinic who were found to have NSVT on ambulatory patch monitors (2017–2023). Among 2805 pts, 172 (age 29 ± 14, range: 3–81) had NSVT yielding a prevalence of 6% and a monitoring duration of 7.5 ± 5.5 days. Cardiac diagnoses included: 57% CHD, 26% structurally normal hearts, 8% cardiomyopathy, 6% connective tissue disorder, and 3% channelopathy. Mean day of NSVT detection was 4.3 ± 3.7 days without significant difference amongst the various underlying cardiac diagnoses. Management changes were made in 52 pts: medication change in 43, loop recorder placement in 4, and cardiac defibrillator placement in 5. Five pts died with 1 suspected sudden cardiac death. The likelihood of NSVT detection increased with longer monitoring duration with only 55% of cases detected at 2 days. In a multivariable model, monitoring duration but not underlying diagnosis, age, monitor indication, or single ventricle status, was associated with the likelihood of NSVT detection. While NSVT is relatively uncommon, it is increasingly detected in pediatric and CHD pts with longer monitoring durations, regardless of the underlying cardiac condition. Although medical management changes based on monitor findings were frequent, sudden death was rare.

**Supplementary Information:**

The online version contains supplementary material available at https://doi.org/10.1007/s00246-025-04086-y.

## Introduction

Nonsustained ventricular tachycardia (NSVT), defined as greater than 3 consecutive ventricular beats at a rate greater than 120 bpm or > 20% of the baseline sinus rate for less than 30 s, is commonly seen in patients with underlying congenital heart disease (CHD) and/or cardiomyopathy (CMP), but is thought to be an uncommon arrhythmia in otherwise healthy children [1, 2]. This arrhythmia is often asymptomatic and typically diagnosed on ambulatory monitors. Extended cardiac ambulatory monitoring devices are now widely available and have the capability of recording continuous single lead electrocardiogram (ECG) data up to 30 days [3, 4]. Therefore, these newer devices provide an opportunity to monitor patients for longer, which could facilitate an increase in the detection rate of asymptomatic arrhythmias, like NSVT. Compared to the routine 24–48 Holter monitor, the use of extended ambulatory monitoring has been shown to improve detection of atrial fibrillation in adults patients with cryptogenic stroke and following pulmonary vein isolation and more recently in hypertrophic cardiomyopathy [5, 6]. Similarly, in a cohort of patients with adult congenital heart disease (ACHD), Schultz et al. reported that ~ 50% of patients had significant arrhythmias detected beyond 48-hour monitoring period [7]. However, the clinical significance of NSVT detected on extended ambulatory monitoring (beyond 48 h) in pediatric patients with structurally normal heart (SNH) or those with CMP has not been thoroughly investigated.

The primary objective of this study is to examine the prevalence of NSVT as documented on extended ambulatory monitoring at our center and to analyze the presence or absence of associated cardiovascular disease. The secondary objective is to determine how the identification of NSVT on extended monitoring affects clinical management. We hypothesize that a significant proportion of NSVT episodes will be detected beyond the 48-hour monitoring period, further supporting the use of extended monitoring in certain patient populations. We also hypothesize that with increased detection of NSVT, there will be a corresponding increase in treatment changes for patients.

## Methods

### Study Design and Study Population

We conducted a single center, retrospective observational cohort study, of all patients evaluated in the outpatient cardiology clinic at Cleveland Clinic Children’s who were found to have NSVT on continuous ambulatory patch monitors (Zio XT, iRhythm Technologies, San Francisco, CA) from January 2017 to November 2023. NSVT was defined as greater than 3 consecutive beats of ventricular tachycardia at a rate greater than 120 bpm or > 20% of the baseline sinus rate and less than 30 s [2]. Patients with wide complex tachycardia determined to be supraventricular tachycardia with aberrancy, as adjudicated by a pediatric electrophysiologist, were excluded. Electronic medical records were reviewed for the following variables: demographic information, related cardiac diagnosis and symptoms, indications for monitor placement, family history of channelopathy or sudden death in the young or CMP, echocardiogram findings (ventricular size and function, presence of mitral valve prolapse and/or mitral annular disjunction), presence of inducible ventricular arrhythmias on exercise stress test (defined as greater than 3 beats of ventricular tachycardia in all patients or couplets or more in patients with catecholaminergic polymorphic ventricular tachycardia), ventricular function and size and presence of scar on cardiac MRI, genetic test results if applicable, and NSVT episodes’ characteristics (number of episodes, rate of NSVT including maximum and average, total number of beats per episode, and day of detection). If a patient had more than one ambulatory monitor showing NSVT, up to three monitors were recorded and the NSVT details on these monitors were analyzed and described in the results section. Patients were categorized based on the severity and complexity of their CHD into simple, moderate, and complex based on the 2018 AHA/ACC guidelines for the management of ACHD patients [8]. Additionally, patients were further classified based on their anatomy and physiology into single ventricle or biventricular circulation. Management changes based on results of the ambulatory monitor were also reviewed and documented and these include medication changes (change in beta blocker dose or type if patient is already on a beta blocker or initiation of a new beta blocker or new antiarrhythmic medication), implantable loop recorder for closer monitoring, implantable cardiac defibrillator (ICD) placement, or electrophysiology (EP) study and catheter ablation. All data were collected and stored in REDCap. Our study was approved by the Cleveland Clinic institutional review board.

### Statistical Analysis

Categorical variables were expressed as numbers and percentages and were compared using Pearson’s χ2 test. Continuous variables were reported as mean with standard deviation or median with their corresponding interquartile ranges. Continuous variables were compared using the independent t-test. Subgroup analysis was performed based on monitor duration, underlying diagnosis, and medication change. Prevalence was calculated by dividing the number of patients that had any ambulatory monitor with NSVT during that time frame by the total number of patients who had ambulatory monitors in that time frame. Kaplan-Meier curves and Cox proportional hazards model were generated for time to NSVT detection. We assessed whether the underlying cardiac diagnosis was an important determinant of the time to NSVT detection. Two adjustments were made: one for monitoring duration as a sole hazard coefficient, and the other for monitoring duration along with other important coefficients such as underlying CHD, CMP, channelopathy, biventricular status, monitoring reason, and age category. Only significant coefficients were included to avoid overfitting. Analyses were performed using Stata (StataCorp. 2016. Stata Statistical Software: Release 14.2. College Station, TX: StataCorp LLC). P value < 0.05 is considered statistically significant.

## Results

### Baseline Cohort Characteristics and NSVT Prevalence

Of 2,805 patients who were evaluated in our outpatient cardiology clinic and underwent ambulatory monitoring using patch devices, 172 had NSVT and were included in our study. Overall NSVT prevalence was 6%. A second monitor was prescribed in 113 patients (66%), of whom 39 (35%) had NSVT. A third monitor was prescribed in 71 patients (41%), of whom 23 (34%) had NSVT. Of the 234 monitors with NSVT, the average day of NSVT detection was 4.3 ± 3.7 days, with an average monitoring duration of 7.5 ± 5.5 days. The average rate of NSVT was 149 ± 34 beats per minute, while the maximum rate was 173 ± 39 beats per minute.

The mean age at NSVT detection was 29 ±$\:\:14\:$years (range: 3–81) with 22% ≤ 18 years of age. Most patients were male (88, 51%). Indications for monitor placement were as follows: symptoms (97, 56%), history of arrhythmias (34, 20%), and screening due to perceived risk of arrhythmias given underlying disease (36, 21%). Cardiac diagnoses included 98 (57%) with CHD, 44 (26%) with SNH, 14 (8%) with CMP, 10 (6%) with connective tissue disorder (CTD), and 6 (3%) with channelopathy. Among those with CHD, 1 was classified as simple CHD, 47 had moderate CHD, and 50 had complex CHD. A total of 25 patients (14%) had single ventricle physiology. The majority of patients had normal ventricular function (137, 80%). Late gadolinium enhancement (LGE) was present in 13 patients (7.6%).

Of the 172 patients, 48 had a monitoring duration of ≤ 48 h and 124 had a monitoring duration of > 48 h. There were no significant differences in baseline characteristics or demographics except that patients with the longer monitoring duration were more likely to be symptomatic and as such were more likely to receive a monitor for symptom evaluation (33% vs. 65%, ≤ 48 h vs. > 48 h, *p* = 0.001). Patient demographics and baseline characteristics including comparisons between the two groups are shown in Table 1.

**Table 1:** Baseline cohort characteristics

	Monitor duration ≤ 48 h (*n* = 48)	Monitor duration > 48 h (*n* = 124)	*P* value
Age, yrs (mean ± SD)	27 ± 14	30 ± 14	0.18
Gender, n (%)			0.38
Male	22 (46)	66 (53)	
Female	26 (54)	58 (47)	
Symptoms, n (%)			0.006
Yes	27 (56)	96 (78)	
No	21 (44)	28 (23)	
Cardiac diagnosis, n (%)			0.19
CHD	32 (67)	66 (53)	
CMP	1 (2)	13 (11)	
Channelopathy	1 (2)	5 (4)	
CTD	1 (2)	9 (7)	
SNH	13 (27)	31 (25)	
CHD, n (%)			0.37
Simple Moderate	0 16 (33)	1 (1) 31 (25)	
Complex	16 (33)	34 (27)	
Ventricular status, n (%)			
Biventricular	23 (48)	50 (40)	0.25
Single ventricle	9 (19)	16 (3)	
Indications for monitor placement, n (%)			0.001
Symptoms	16 (33)	81 (65)	
Hx of arrhythmias	12 (25)	22 (18)	
Screening	18 (38)	18 (15)	
Other	2 (4)	3 (2)	
# of NSVT episodes on 1st monitor, median (range)	1 (1,12912)	1 (1,6171)	
Echocardiogram, n (%)			0.55
Normal	40 (83)	97 (80)	
Mild dysfunction	3 (6)	15 (12)	
Moderate dysfunction	1 (2)	4 (3)	
Severe dysfunction	4 (8)	6 (5)	
Medication changes	8 (17)	35 (28)	0.12
Management changes			
EP study	0	1 (1)	1.00
ILR placement	0	4 (3)	0.58
ICD placement	0	5 (4)	0.32

*CHD* congenital heart disease, *SNH* structurally normal heart, *CTD* connective tissue disease, *CMP* cardiomyopathy, *DCM* dilated cardiomyopathy, *HCM* hypertrophic cardiomyopathy, *MVP* mitral valve prolapse, *MAD* mitral annular disjunction, *EST* exercise stress testing, *LGE* late gadolinium enhancement

### Association between NSVT and Underlying Cardiac Diagnoses

Table 2 shows NSVT data by underlying diagnosis. When comparing the various underlying cardiac conditions, our analysis showed no statistically significant differences in NSVT characteristics, such as the timing of detection, frequency of episodes, or NSVT rates.

**Table 2:** NSVT data by underlying diagnosis

	SNH	CHD	CTD	CMP	CHN	*P* Value
Day of NSVT detection, mean ± SD	4.7 ± 3.8	4.2 ± 3.5	6.8 ± 4.6	3.6 ± 2.6	5.3 ± 4.2	0.20
# of NSVT episodes, median (range)	1 (1,12912)	1 (1,6171)	1 (1,2)	1 (1,15)	4.5 (1, 30)	0.50
NSVT rate, mean ± SD	147 ± 36	148 ± 32	168 ± 38	144 ± 24	165 ± 33	0.26
Maximum NSVT rate, mean ± SD	174 ± 40	171 ± 32	190 ± 33	164 ± 30	207 ± 53	0.06
NSVT on f/u, n (%)	7 (29%)	35 (51%)	5 (50%)	2 (29%)	0	0.16
# of NSVT episodes on 2nd f/u, median (range)	7 (1, 3966)	1 (1,12062)	4 (1,4)	7.5 (1,14)	-	0.64
# of NSVT on 3rd f/u, median (range)	5293 (1,10586)	1 (1,250)	2 (1,3)	-	-	0.78
NSVT rate on f/u, mean ± SD	159 ± 39	151 ± 35	162 ± 31	136 ± 16	0	0.81
Maximum NSVT rate on f/u, mean ± SD	184 ± 42	173 ± 41	178 ± 30	151 ± 22	0	0.77

*SNH* structurally normal heart, *CHD* congenital heart disease, *CMP* cardiomyopathy, *CTD* connective tissue disease, *CHN* channelopathy

### Factors Associated with time To NSVT Detection

**Fig. 1:**
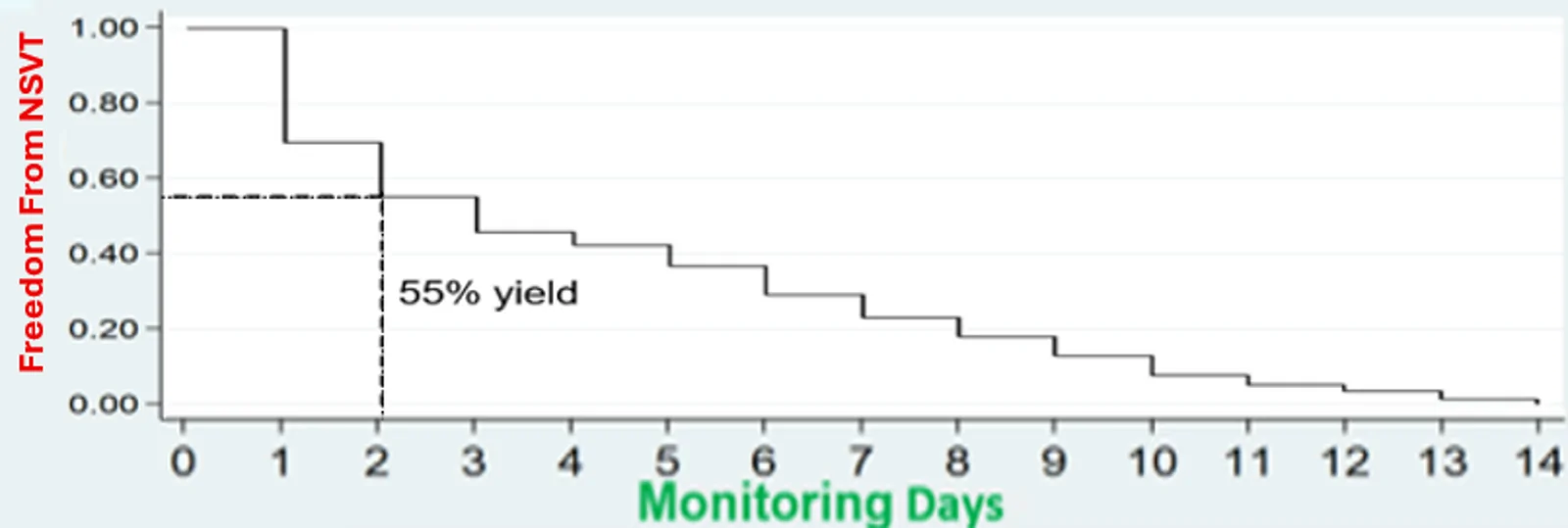
Kaplan Meier curve demonstrating time to non-sustained ventricular tachycardia (NSVT) detection on ambulatory monitoring

**Fig. 2:**
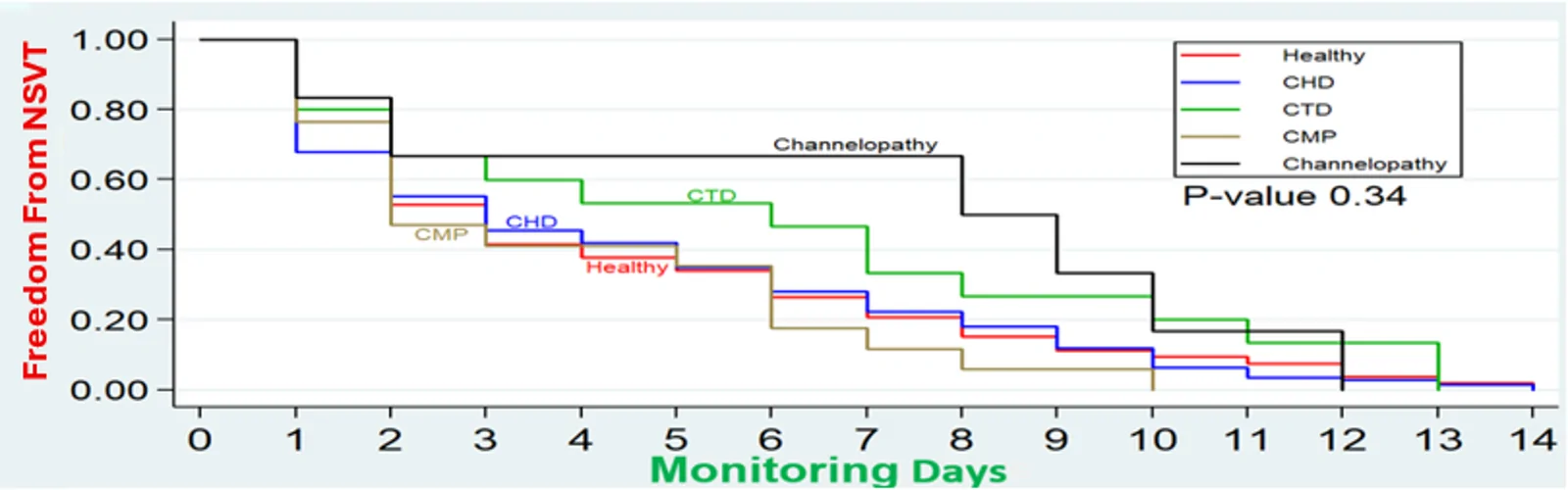
Kaplan Meier curve demonstrating time to non-sustained ventricular tachycardia (NSVT) detection on ambulatory monitoring by underlying cardiac diagnosis

**Fig. 3:**
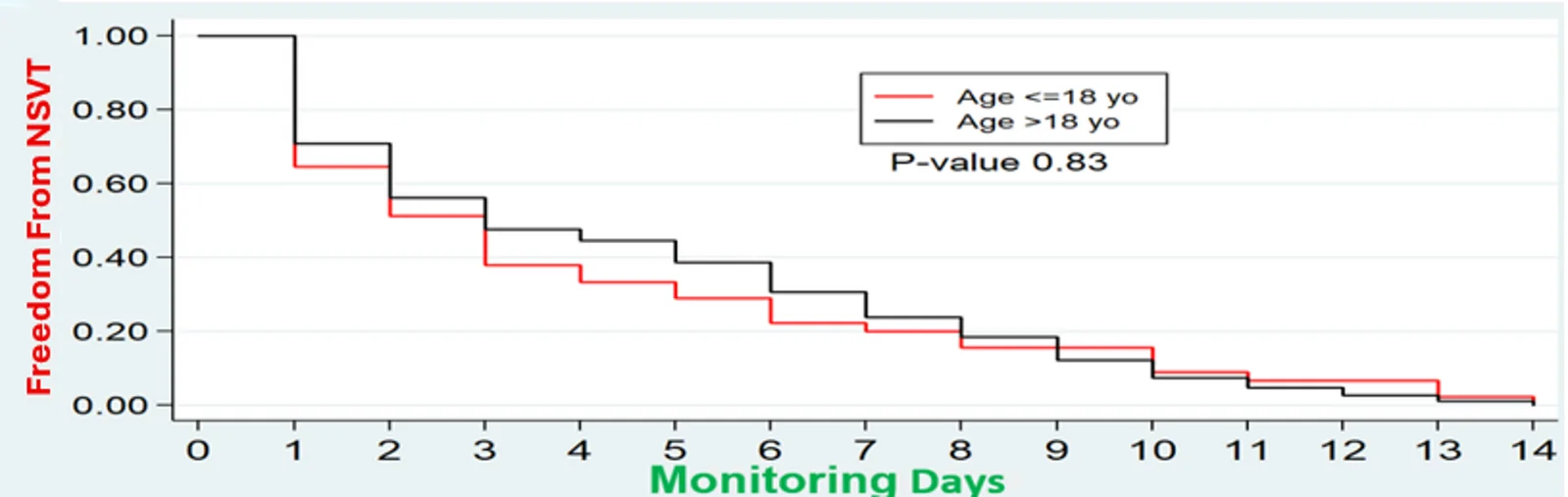
Kaplan Meier curve demonstrating time to non-sustained ventricular tachycardia (NSVT) detection on ambulatory monitoring by age

**Fig. 4:**
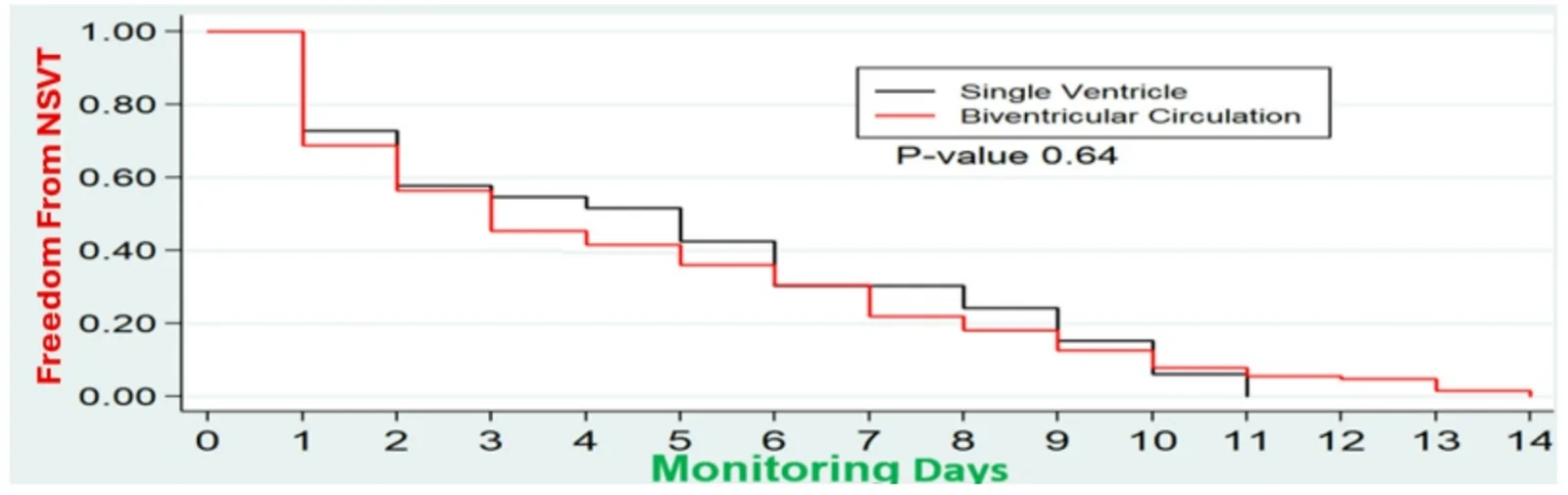
Kaplan Meier curve demonstrating time to non-sustained ventricular tachycardia (NSVT) detection on ambulatory monitoring by ventricular status

Using a Kaplan-Meier curve analysis, our data showed that 25% of NSVT cases were detected within 1 day, 50% within 3 days, and 75% within 7 days of monitoring, Fig. 1. In a multivariable Cox regression model adjusting for monitoring duration, patients with CHD had an 8% higher hazard of NSVT detection compared to those without CHD; however, this difference was not statistically significant (HR = 1.08; 95% CI: 0.83–1.41; *p* = 0.572), Fig. 2. Similarly, there was no statistically significant difference in NSVT detection between adult (age > 18 years) and pediatric (age ≤ 18 years) patients (HR = 0.96; 95% CI: 0.69–1.34; *p* = 0.828), Fig. 3, or between patients with biventricular and single-ventricle circulation (HR = 0.91; 95% CI: 0.62–1.34; *p* = 0.640), Fig. 4.

In all tested models, monitoring duration was strongly and independently associated with the outcome. In a multivariable Cox proportional hazards model adjusting for all the previous covariates, each additional day of monitoring was associated with a statistically significant 20% decrease in the hazard of detecting NSVT (HR = 0.80; 95% CI: 0.77–0.84; *p* < 0.001) signifying that rate of NSVT detection decreased as time went by but overall cumulative NSVT detection rate increased with monitoring duration.

### Changes in Management

The monitor findings led to medication changes in 43 patients (25%), 35 of whom belong to the > 48 h monitoring period. Table 3 shows characteristics of patients with and without medication changes. Patients with medication changes were more likely to have CHD (70% vs. 53%, *p* = 0.06) and less likely to have SNH (9% vs. 31%, *p* = 0.06) but that did not reach statistical significance. Patients with medication changes were more likely to have NSVT on follow-up monitors (63% vs. 31%, *p* = 0.001). There was a trend towards a longer follow-up monitor duration in those with medication changes vs. those without (8.1 ± 5.5 vs. 6.0 ± 5.5 years, *p* = 0.063), supplementary Table 1. The specific medication changes can be found in supplementary Table 2.

**Table 3:** Comparison of patients with and without medication changes

	Patients with no medication change (*n* = 129)	Patients with medication change (*n* = 43)	*P* value
Age, yrs (mean ± SD)	30 ± 15	29 ± 12	0.61
Gender, n (%)			0.08
Male	61 (47)	27 (63)	
Female	68 (53)	16 (37)	
Cardiac symptoms			0.25
Chest pain	4 (3)	4 (9)	
Cardiac syncope	1 (1)	2 (5)	
Vasovagal syncope	2 (2)	0	
Palpitations	47 (36)	16 (37)	
> 1 symptom None	37 (29) 38 (30)	10 (23) 11 (26)	
Symptoms, n (%)			0.55
Yes	93 (72)	33 (77)	
No	36 (28)	10 (23)	
Cardiac diagnosis, n (%)			0.06
CHD	68 (53)	30 (70)	
CMP	11 (9)	3 (7)	
Channelopathy	4 (3)	2 (5)	
CTD	6 (5)	4 (9)	
SNH	40 (31)	4 (9)	
CHD, n (%)			0.22
Simple	1 (1)	0	
Moderate	32 (25)	15 (35)	
Complex	35 (27)	15 (35)	
Ventricular status, n (%)			0.14
Biventricular	50 (39)	23 (54)	
Single ventricle	18 (14)	7 (16)	
Monitor indication, n (%)			0.71
Symptoms	71 (55)	26 (61)	
Hx of arrhythmias	28 (22)	6 (14)	
Screening	26 (20)	10 (23)	
Other	4 (3)	1 (2)	
Echocardiogram, n (%)			0.82
Normal function	102 (80)	35 (83)	
Mild dysfunction	15 (12)	3 (7)	
Moderate dysfunction	4 (3)	1 (2)	
Severe dysfunction	7 (6)	3 (7)	
EST testing, n (%)			
Inducible arrhythmia	0	1 (3)	0.31
Day when NSVT was detected	4.3 ± 3.7	5.2 ± 3.8	0.16
# of NSVT episodes, median (range)	1 (1, 12912)	1 (1, 6171)	0.31
NSVT rate, mean ± SD	148 ± 33	151 ± 34	0.67
Maximum NSVT rate, mean ± SD	173 ± 36	175 ± 36	0.70
Presence of NSVT on f/u, n (%)	25 (34)	24 (60)	0.08
# of NSVT episodes on 2nd f/u, median (range)	1 (1, 3966)	4 (1, 12062)	0.28
# of NSVT episodes on 3rd f/u, median (range)	1 (1,10586)	1 (1, 250)	1.00
NSVT rate on f/u, mean ± SD	153 ± 39	152 ± 28	0.96
Maximum NSVT rate on f/u, mean ± SD	176 ± 46	173 ± 31	0.83
Monitor Duration, n (%)			0.12
<48 h	40 (31)	8 (19)	
>48 h	89 (69)	35 (81)	
Presence of LGE, n (%)	10 (8)	3 (7)	1.00

*CHD* congenital heart disease, *SNH* structurally normal heart, *CMP* cardiomyopathy, *EST* exercise stress testing

Additional management changes were as follows: 4 underwent ILR placement for close monitoring, and 5 had ICD placement. All 9 patients belong to the > 48 h monitoring group (Table 1). Five patients underwent ICD placement for primary prevention: 3 with HCM, 1 with D-transposition of the great arteries status post Mustard procedure, and 1 with L-transposition of the great arteries status post double switch repair. Among the ILR patients, 1 had dilated CMP, 1 had runs of idiopathic ventricular tachycardia of unclear etiology, 1 had Marfan syndrome, and 1 had NSVT with a family history of HCM.

### Patient Outcomes

Of the total cohort, 5 patients died during the study period with only 1 suspected sudden cardiac death. This patient was a young female with repaired tetralogy of Fallot with no identifiable risk factors for SCD other than NSVT on ambulatory monitoring. She passed away in her sleep, and no autopsy was performed to assess for other potential causes of death.

## Discussion

The goal of this study was to assess NSVT prevalence in a large pediatric and ACHD cohort, identify factors associated with NSVT detection, and assess management changes. The main findings in this study are as follows: (1) overall NSVT prevalence on ambulatory monitors is 6%; (2) longer monitoring durations resulted in higher detection rate and were not impacted by patient characteristics or diagnoses; (3) management changes were frequent in our cohort, but SCD was rare.

The literature on NSVT prevalence in pediatrics and CHD is scarce as most studies focus on sustained VT/VF. Additionally, the published prevalence of NSVT in this patient population has varied widely between 11% and 33% depending on screening methodology, studied population, and monitoring duration [7, 9, 10]. In a retrospective single center cohort of repaired ACHD patients excluding those with tetralogy of Fallot, device detected NSVT was reported at 33% of the studied population [9]. In another study, Schultz et al. demonstrated that NSVT was the second most common arrhythmia detected on ambulatory monitoring in ACHD patients with an overall prevalence of 11% [7]. The lower prevalence in our cohort is likely attributed to differences in studied patient population as our cohort included not only ACHD patients but also pediatric patients. A variety of underlying cardiac conditions were present in our study cohort, including CHDs, CMP, CTDs, SNH, and channelopathies.

Another important finding of our study is that longer monitoring duration resulted in higher detection of NSVT rates with only 55% of cases detected by 48 h, which is currently the recommended monitoring duration per published US guidelines for CHD [8]. Our data showed that irrespective of underlying diagnosis, age, ventricular circulation status, and monitoring indication, monitoring duration is the sole predictor of likelihood of NSVT detection. Additionally, at least 7 days of monitoring were required to capture 75% of the episodes. Concordant with our findings, Schultz et al. demonstrated that arrhythmia detection was higher in an ACHD cohort who underwent extended ambulatory monitoring for 14 days vs. 48 h only [7]. In fact, only 46% of captured arrhythmias were found in the first 48 h. The only difference between those monitored for < 48 h vs. > 48 h in our study cohort was indication for ambulatory monitor. Patients with symptoms as the primary indication were more likely to be monitored for longer duration than those with history of previous arrhythmias or those who received it for screening for occult arrhythmias. This is intuitive as in these cases correlating patient symptoms with ambulatory rhythm findings is key in identifying the clinical diagnosis, which would inform clinical practice.

An important question that our study addressed is the impact of NSVT detection on clinical management. Although NSVT is an established risk factor for VT and SCD in HCM and tetralogy of Fallot patients [11, 12], little is known about its role in otherwise healthy individuals or those with other forms of CHD. Doctor et al. noted a significant association between device detected NSVT and the primary outcome of VT/VF or SCD in a cohort of adults with CHD after excluding those with tetralogy of Fallot [9]. It is important to note that patients with NSVT were older at time of device implant, had systemic ventricular dysfunction, and history of ventriculotomy. These are important risk factors that put patients at higher risk. Based on the significant differences between the two groups, the authors caution the reader that NSVT should not be used as an independent risk factor for SCD in a heterogenous population. In another study and concordant with our study findings, the authors found that NSVT is common in ACHD patients, and these patients rarely developed sustained VT/VF leading them to recommend a “wait and see” treatment strategy [13]. Giacone et al. showed that ventricular arrhythmias were highly associated with transplant or death in Fontan patients; however, their study included both sustained and non-sustained VT, with the former being a more clinically significant arrhythmia [10]. On the contrary, Czosek et al. showed that the presence of NSVT on Holter monitor was not associated with outcomes in Fontan patients or those with transposition of the great arteries [14].

In our study, management changes were frequent and occurred in one third of the study cohort. Most of the management changes constituted medication change as well as a considerable proportion received primary prevention ICDs or ILRs for closer monitoring. Interestingly, the detection of NSVT on follow-up monitors was notably higher in patients who underwent medication changescompared to those who did not This can be partly explained by the longer monitoring duration on follow up monitors and may reflect a higher-risk population as perceived by the primary cardiologist. The presence of CHD rather than specific NSVT characteristics (i.e. number of episodes, rate of NSVT, duration of NSVT) showed trend toward influencing the decision of the primary physician to change the patient management plan. The other interesting finding was that all 9 patients who underwent device placement and 82% of those with medication changes had NSVT detected beyond the initial 48-hour monitoring period, highlighting the impact of these detected arrhythmias on patient clinical management. Despite the 6% prevalence of NSVT in our diverse study cohort and the frequent management changes, SCD or death related to VT/VF was uncommon.

## Limitations

While our study provides valuable insights, certain limitations must be acknowledged. The retrospective nature of the study may introduce selection bias and limit the ability to infer causality. It is important to mention that changes were made at the discretion of the primary physician and other clinical factors beyond NSVT detection likely impacted decision making. As this study was conducted at a single center, the findings may not be generalizable to other populations. Differences in monitoring durations among patients could influence the detection rates and characteristics of NSVT, potentially introducing bias. Future research should focus on conducting prospective, multicenter studies to validate our findings with a focus on investigating optimal monitoring durations for various patient subgroups and evaluating the long-term outcomes of patients with NSVT detected by ambulatory monitors.

## Conclusion

While NSVT is relatively uncommon, it is increasingly detected in pediatric and CHD patients with longer monitoring durations, regardless of the underlying cardiac condition. The high rate of NSVT detection beyond the “typical” 48-hour monitoring period underscores the necessity of extended monitoring to capture arrhythmic events and/or substrates that may prove to be significant predictors of arrhythmias in the future. Medical management changes based on monitor findings were frequent but sudden death was rare. The clinical significance of these findings requires further prospective studies.

## Supplementary Information

Below is the link to the electronic supplementary material.

Supplementary file 1 (DOCX 22 KB)

## Data Availability

No datasets were generated or analysed during the current study.

## Abbreviations

NSVT: Non-sustained ventricular tachycardia
ACHD: Adult congenital heart disease
CHD: Congenital heart disease
CMP: Cardiomyopathy
CTD: Connective tissue disorder
SNH: Structurally normal heart
