# Supplementary material for: Non-sustained Ventricular Tachycardia in Children and Adult Congenital Heart Disease: Optimal Duration and Clinical Significance
Source: Pediatr Cardiol. 2025 Nov 13;47(7):3034–42. doi: 10.1007/s00246-025-04086-y (PMC13597579; doi:10.1007/s00246-025-04086-y)
Supplement: Supplementary file 1 [file 246_2025_4086_MOESM1_ESM.docx]

| Supplementary Table 1: Follow-up monitor after medication change vs no medication change | | | |
| --- | --- | --- | --- |
|  | **Patients with no medication change**  **(n = 129)** | **Patients with medication change**  **(n = 43)** | **P value** |
| Follow-up monitor, n(%) | 73 (57) | 39 (91) | < 0.001 |
| Follow-up monitor duration, mean ± SD | 6.0 ± 5.5 | 8.1 ± 5.5 | 0.063 |
| Monitoring duration (> 48 hrs), n (%) | 34 (47) | 25 (64) | 0.08 |

| Supplementary Table 2: Characteristics of Medication Changes | |
| --- | --- |
|  | **Number of patients**  **(n = 43)** |
| Type of change, n (%)  New medication  Change in medication  Dose change | 28 (65)  6 (14)  9 (21) |
| New medication, n (%)  Nadolol  Carvedilol  Metoprolol  Propranolol  Atenolol  Verapamil  Flecainide | 8 (28)  6 (21)  10 (35)  1 (4)  1 (4)  1 (4)  1 (4) |
| Change in medication, n (%)  Metoprolol to Nadolol  Verapamil to Metoprolol  Nadolol to Flecainide  Atenolol to Metoprolol  Carvedilol to Sotalol  Atenolol to Nadolol | 1 (17)  1 (17)  1 (17)  1 (17)  1 (17)  1 (17) |
| Dose change, n (%)  Nadolol  Carvedilol  Metoprolol | 6 (67)  1 (11)  2 (22) |
